# Decitabine immunosensitizes human gliomas to NY-ESO-1 specific T lymphocyte targeting through the Fas/Fas Ligand pathway

**DOI:** 10.1186/1479-5876-9-192

**Published:** 2011-11-07

**Authors:** Veerauo V Konkankit, Won Kim, Richard C Koya, Ascia Eskin, Mai-Anh Dam, Stanley Nelson, Antoni Ribas, Linda M Liau, Robert M Prins

**Affiliations:** 1 Graduate Program in Physiological Sciences, David Geffen School of Medicine at UCLA, University of California Los Angeles, Los Angeles, California, 90095, USA; 2 Department of Neurosurgery, David Geffen School of Medicine at UCLA, University of California Los Angeles, Los Angeles, California, 90095, USA; 3 Surgery/Surgical Oncology, David Geffen School of Medicine at UCLA, University of California Los Angeles, Los Angeles, California, 90095, USA; 4 Medicine/Hematology-Oncology, David Geffen School of Medicine at UCLA, University of California Los Angeles, Los Angeles, California, 90095, USA; 5 Human Genetics, David Geffen School of Medicine at UCLA, University of California Los Angeles, Los Angeles, California, 90095, USA; 6 Jonsson Comprehensive Cancer Center, David Geffen School of Medicine at UCLA, University of California Los Angeles, Los Angeles, California, 90095, USA; 7 Brain Research Institute, David Geffen School of Medicine at UCLA, University of California Los Angeles, Los Angeles, California, 90095, USA; 8 Institute for Molecular Medicine, David Geffen School of Medicine at UCLA, University of California Los Angeles, Los Angeles, California, 90095, USA

**Keywords:** T cells, tumor immunity, decitabine, cancer testis antigens, Fas/Fas Ligand

## Abstract

**Background:**

The lack of effective treatments for gliomas makes them a significant health problem and highlights the need for the development of novel and innovative treatment approaches. Immunotherapy is an appealing strategy because of the potential ability for immune cells to traffic to and destroy infiltrating tumor cells. However, the absence of well-characterized, highly immunogenic tumor-rejection antigens (TRA) in gliomas has limited the implementation of targeted immune-based therapies.

**Methods:**

We hypothesized that treatment with the demethylating agent, decitabine, would upregulate the expression of TRA on tumor cells, thereby facilitating enhanced surveillance by TRA-specific T cells.

**Results and Discussion:**

Treatment of human glioma cells with decitabine increased the expression of NY-ESO-1 and other well characterized cancer testes antigens. The upregulation of NY-ESO-1 made these tumors susceptible to NY-ESO-1-specific T-cell recognition and lysis. Interestingly, decitabine treatment of T98 glioma cells also sensitized them to Fas-dependent apoptosis with an agonistic antibody, while a Fas blocking antibody could largely prevent the enhanced functional recognition by NY-ESO-1 specific T cells. Thus, decitabine treatment transformed a non-immunogenic glioma cell into an immunogenic target that was efficiently recognized by NY-ESO-1--specific T cells.

**Conclusions:**

Such data supports the hypothesis that agents which alter epigenetic cellular processes may "immunosensitize" tumor cells to tumor-specific T cell-mediated lysis.

## Background

Glioblastoma is the most malignant of the glial tumors. Patients with glioblastoma have a 5-year survival rate of less than 3.3% despite advances in surgery, radiation, and chemotherapeutic techniques [1-3]. Immunotherapy is a promising new treatment strategy for brain tumors that may allow for stimulation of an antitumor immune response while sparing normal brain structures [4,5]. However, gliomas lack well defined TRA for immune targeting by CD8^+ ^T-cell effectors.

Cancer testis antigens (CTAs) are expressed in a variety of tumors, but not in non-neoplastic normal human cells, with the exception of germ cells [6,7]. NY-ESO-1, a member of the cancer/testis antigen family, is considered to be one of the most immunogenic TRAs and therefore a promising target for cell mediated immunity [8-13]. NY-ESO-1 expression has been identified at the protein level in a variety of malignant neoplasms such as lung cancers, melanomas, synovial sarcomas, and bladder cancers [11,13-15]. Previous studies, however, have demonstrated low levels of NY-ESO-1 detected at the mRNA level in brain tumors [14,16]. Recent evidence has suggested that compounds that influence DNA methylation can also up-regulate the expression of CTAs [17,18]. This increase in gene expression appears to increase the antigenicity of CTA in some cancers, such as gliomas, myeloid leukemia and renal cell carcinoma [19-23].

Decitabine (5-aza-2'-deoxycytidine) is a cytosine analogue that incorporates itself into the DNA strands of proliferating cells [24-26]. It effectively inhibits DNA methylation and increases gene expression by covalently binding to the promoter regions of DNA methyltransferase [24]. DNA methylation is also critically involved in embryonic development, transcription, chromatin structure, X-chromosome inactivation, genomic imprinting, and chromosome stability [27,28]. Aberrant hypermethylation represses transcription by way of CpG islands in the promoter region and is associated with gene inactivation. Use of decitabine induces re-expression of certain genes that are otherwise repressed in cell culture [24]. We hypothesized that treatment with decitabine would up-regulate the expression of CTAs in gliomas, thereby sensitizing human glioma cells to immune-based therapies without having similar effects upon normal cells.

A recent study provided the first evidence in a glioma model that decitabine treatment could increase the expression of NY-ESO-1 and other CTA [21]. In this study, we analyzed the expression of NY-ESO-1 in 5 established human glioma cell lines and 4 primary glioma cell lines. We demonstrated that their treatment with decitabine up-regulated the expression of NY-ESO-1 and MHC I class, and as a result, induced simultaneous release of T cell effector pro-inflammatory cytokines and tumor cell killing. These results suggest that treatment with decitabine not only increases the expression of an immunogenic CTA, but can also re-establish functionality of the apoptotic signaling system within tumor cells and sensitize these cells to immune-mediated cell death via the Fas/Fas Ligand pathway.

## Methods

### Cell lines and human brain tissue samples

Five cell lines, DBTRG05-MG, SNB-19, U-251MG, U-373MG and T98G derived human glioblastoma cell lines were kindly provided by Dr. Carol Kruse (UCLA Department of Neurosurgery, Los Angeles, CA). They were maintained in DMEM (Mediatech, Inc., Herndon, Virginia) supplemented with 10% FBS (Gemini Bio-Products, West Sacramento, California), 1% (v/v) penicillin, streptomycin, and amphotericin B (Mediatech Cellgro, Manassas, Virginia) and kept in an atmosphere of 5% CO_2 _at 37°C. Normal human astrocytes (NHA) were kindly provided by Dr. Russ Pieper (UCSF Department of Neurosurgery, San Francisco, CA). A melanoma cell line, 624.38, was kindly provided by Dr. Steve Rosenberg (NIH/NCI). Both NHA and 624.38 cells were placed into identical culture conditions to glioblastoma cell lines.

Primary tumor cell cultures were derived from four patients with glioblastomas who had undergone surgical resection at the UCLA Medical Center. These tissues were cultured by digesting homogenized tumors in a collagenase DNAse mixture overnight as previously described [5,29]. The digested tumor samples were filtered through a mesh and the cells placed into complete medium as described earlier. The cells were incubated in 5% CO_2 _at 37°C. Human brain tissue samples were also obtained from patients who had undergone resection at the UCLA Medical Center, CA. All patients provided written informed consent for these procedures.

Peripheral blood was obtained from healthy human volunteers provided by the Division of Hematology and Oncology at UCLA Medical Center, Los Angeles. Peripheral blood mononuclear cells (PBMC) were isolated by Ficoll gradient centrifugation as previously described [5]. Written informed consent and institutional IRB approval was obtained for all studies involving human bloods and tissues.

### Reagent

Decitabine (5-aza-2'-deoxycytidine) was generously supplied by Eisai Pharmaceuticals (Woodcliff Lake, New Jersey). A 10 μM stock solution of it in DMSO was stored at -80°C.

### In vitro treatment of cultured cells with decitabine

Cells were plated overnight at 10^6^cells/ml at 37°C in a 5% CO_2 _incubator. The following day, the cell culture medium was replaced with either fresh medium or that supplemented with 1 μM decitabine. The cells were treated again the following day in new cell culture medium. At the end of treatment, the medium was replaced with fresh medium without decitabine, and the cells were cultured for an additional 48 hours before utilized in subsequent assays.

### Conventional reverse transcription PCR analysis of NY-ESO-1 expression

Total RNA was isolated with the RNeasy Mini kit (Qiagen, Valencia, California) according to manufacturer's protocol. 3 × 10^6 ^human glioblastoma cancer cell lines or 25 mg of tumor tissue samples were used. cDNA was synthesized from 1 μg total RNA by using Omniscript Reverse Transcription kit (Qiagen), again according to the manufacturer's protocol. PCR was facilitated by using the Accuprime GC-rich kit (Invitrogen, Carlsbad, California). In each PCR, 2 μl cDNA was used in a 35-μl reaction volume containing 10 μM sense and antisense primers, 5 μL 5× PCR buffer (A for GC-rich genomic DNA target or B for non-GC-rich genomic DNA target), and 1 μl of Accuprime GC-Rich DNA polymerase (Invitrogen). The PCR primers were as follows: NY-ESO-1 forward primer: 5'-CATCACGGATCCATGCAGGCCGAAGGCCGG-3', reverse primer: 5'-ACCCGGGGTACCGCGCCTCTGCCCTGAGGG-3', GAPDH forward primer: 5'-GAAGGTGAAGGTCGGAGT-3', reverse primer: 5'-GAAGATGGTGATGGGATTTC-3' (Invitrogen). The PCR cycling parameters were as follows: denaturation at 95°C for 30 s, primer annealing at 60°C for 30 s, and 40 cycles of extension at 72°C for 60 s. PCR cycling was preceded by an initial denaturation at 95°C for 3 min, followed by final extension at 72°C for 10 min. The PCR products were electrophoresed on 1% agarose gels and analyzed after staining with ethidium bromide. Densitometry analysis was performed using the QuantityOne program (BioRad, Hercules, CA).

### Quantitative real-time analysis of NY-ESO-1 expression

Quantitative RT-PCR was performed with the LightCycler real-time RT-PCR system (Roche, Mannheim, Germany), using the LightCycler SYBR green mastermix (Roche). Primers specific for NY-ESO-1 were as follows: forward primer: 5'-TGTCCGGCAACATACTGACT-3', reverse primer: 5'-ACTGCGTGATCCACA TCAAC-3'. GAPDH forward primer: 5'-AGCCACATCGCTCAGACAC-3', reverse primer: 5'-CGCCCAATACGACCAAATC-3' (Invitrogen). Each sample was amplified as follows: 1 cycle at 95°C for 10 min, 40 cycles at 95°C for 15 s, and 60°C for 1 min. Triplicate samples were tested.

### Microarray analysis

Total RNA was extracted from decitabine treated and untreated U-251 MG cells using the RNeasy mini kit (Qiagen). cDNA was generated, quantified and hybridized to U133 Plus 2.0 arrays at the UCLA DNA Microarray Facility using standard Affymetrix protocols. CEL files were normalized using GeneSpring GX 11.5.1 software (Agilent Technologies) with LiWong. To evaluate the baseline expression of NY-ESO-1 in heterogeneous human brain tumor tissues, we analyzed the relative expression of NY-ESO-1 using microarray gene expression profiling in over 300 human brain tumor samples. The relative expression of NY-ESO-1 was normalized and tested. Dots plotted underneath the black line are considered to have at or under background relative expression levels.

### FACS analysis and antibodies

Cells (10^5^) obtained from decitabine-treated and untreated U-251 MG cells were used for flow cytometric analysis. Cells were washed twice with FACS staining buffer and stained with antibodies to HLA-A, B, C (clone DX17) and HLA-A2 (clone BB7.2) (BD Pharmingen, San Diego, California). A Becton Dickinson LSRII was used for acquisition and the acquired data were analyzed using FlowJo (TreeStar, Ashland, Oregon). Gates were set based on singly-stained isotype-control antibodies (data not shown).

### Generation and maintenance NY-ESO-1 TCR-transduced lymphocytes

Six well cluster plates were coated with Retronectin (RN) (Takara, Madison, Wisconsin) at a concentration of 10 μg/ml overnight at 4°C. The next day, blocking buffer (PBS containing 2% Human Serum Albumin) was added for 30 min and washed. Supernates were retrieved from a stable PG13-based retroviral producer cell line encoding a HLA-A*0201-restricted NY-ESO_157-165 _specific T cell receptor (TCR) generated in the MSGV1 retroviral vector backbone, as described [30]. The PG13-based NY-ESO-1 stable retroviral packaging cell line was obtained from Dr. Paul Robbins (Surgery Branch, NCI/NIH). Anti-CD3 (clone OKT-3; 50 ng/ml) was used to stimulate human PBMCs for 48 hours in X-VIVO 15 medium supplemented with 5% human AB serum in the presence of 300 IU IL-2/ml prior to spin-infection. Stimulated PBMCs were transduced twice as described [15,30]. Transduced T cells were maintained in X-VIVO 15 medium supplemented 5% human AB serum and 300 IU IL-2/ml. The cells were expanded for 3 days in the presence of IL-2 and then rested for 2 days in the absence of IL-2.

Transduction efficiency was evaluated 48 hr post-transduction by NY-ESO-1 T cell receptor staining of the CD3^+^CD8^+ ^gated population using antihuman antibodies to CD3-PerCP (clone SK7; BD Biosciences, San Diego, CA), CD8-FITC (clone SFCI21Thy2D3; Beckman Coulter, Brea, CA), and TCR Vβ13.1-PE (clone IMMU 222; Beckman Coulter). Detection of the NY-ESO-1 peptide-MHC complex was accomplished by NY-ESO-1 tetramer staining as described [31].

### CD107A marker staining of NY-ESO-1 TCR transduced PBMC after co-culture with human glioma cells

NY-ESO-1 TCR-transduced PBMCs and untransduced PBMCs were harvested and resuspended at a concentration of 1 × 10^7 ^cells/ml. Treated and untreated HLA-0201^+ ^T98G glioma cells were harvested using trypsin and reconstituted to 1 × 10^7 ^cells/ml. 1 × 10^6 ^PBMCs were co-incubated with target T98G cells in 96-well round-bottomed plates at a 1:1 ratio with total medium volume of 200 μl. Golgi plug (1×) and 5 μl of CD107A-AF647 (clone H4A3) (BD Biosciences) were added into each well and incubated for 6 hr at 37°C. Cells were washed twice with FACS staining buffer (PBS + 2% FBS) and stained with surface antibodies to CD4-PE (clone RPA-T4; BD Biosciences), CD8-PacBlue (clone RPA-T8; BD Biosciences), and CD3-PE-Cy5 (clone UCHT1; BD Biosciences) on ice for 25 min. Cells were washed, fixed with Fixation Buffer (eBioscience, San Diego, CA), and kept at 4°C until analysis. Data were collected using a Becton Dickinson LSRII and analyzed with FlowJo software (TreeStar).

### ELISA and Cytokine Bead Array

To determine cytokine concentrations, supernates were collected from the Golgi-plug free overnight co-cultures. Cell-free supernates were analyzed for interferon-γ using an ELISA kit (eBiosciences) or for simultaneous levels of IFN-γ, TNF-α, IL-2, IL-4, IL-5, and IL-10 using a cytokine bead array (CBA) system (BD Biosciences), following the manufacturer's instructions. Co-cultures at 1:1 (E:T) ratios were placed into complete medium (X-VIVO 15 with 5% human AB serum) in 96-well round-bottomed plates and incubated overnight at 37°C. All samples were in triplicate and supernates were harvested for cytokine detection. IFN-γ ELISAs were performed according to the manufacturer's specification (ELISA Ready-set-go, eBioscience). Color development was stopped after 2-5 minutes. The colorimetric density of each well was measured at 450 nm by a plate reader, and the final concentration of each sample was calculated (pg/ml) based on the standard curve. Triplicate assays were performed for each case and the results were expressed as the mean of 3 separate assays.

Data acquisition for CBA experiments was performed with the FCAP Array software (BD). Once the FCAP Array experiment was created, the files were exported to the BD FACSArray software for sample acquisition and analysis following manufacturer's protocol.

### Apoptosis studies

Apoptosis was induced in T98G glioma cells or 624.38 melanoma cells by the addition of an agonistic anti-CD95 antibody (clone CH-11; Medical and Biological Laboratories). Cells were analyzed for Fas-expression using a CD95-PE mAb (clone DX2) (BD Pharmigen). On the day of harvest, the target cells were cultured in DMEM with FBS containing 200 ng/ml of CH-11 for 24 hr. Cells were washed and resuspended in 1× binding buffer (Invitrogen). Cells were then stained with FITC-conjugated Annexin V (Invitrogen) and the vital dye, propidium iodide (PI, Invitrogen), for 20 min at 37°C. Cells negative for both PI and Annexin V staining were considered live cells; early apoptotic cells were PI-negative, Annexin V-positive; and late apoptotic/dead cells were PI-positive, Annexin V-positive. Each specimen was analyzed in triplicate, and the results shown were the mean ± SE of 3 assays.

### Mixed lymphocyte tumor cell cultures stimulated with antagonistic Fas mAb

PBMCs transduced with the NY-ESO-1 TCR were co-cultured with tumor target cells and blocked with an antagonistic anti-CD95 antibodies (clone ZB4; Medical and Biological Laboratories). Treated and untreated HLA-0201^+ ^T98G glioma cells were harvested using trypsin and reconstituted to 4 × 10^5 ^cells/ml and blocked with antagonistic anti-CD95 antibodies for 1 hr on ice. Cells were washed and reconstituted to 1 × 10^7 ^cells/ml in complete medium. Transduced PBMCs were plated with target cells in 96-well round-bottomed plates at a 1:1 ratio with total medium volume of 200 μl. Golgi plug (1×) and 5 μl of CD107A-AF647 (clone H4A3) (BD Biosciences) were added into each well and incubated for 6 hr at 37°C. Cells were washed twice with FACS staining buffer and stained with surface antibodies to CD4-PE, CD8-PacBlue, and CD3-PE-Cy5 on ice for 25 min. Cells were fixed with Fixation Buffer and kept at 4°C until analysis. Data were collected using a Becton Dickinson LSRII and analyzed using FlowJo software (TreeStar).

### Statistical analysis

Continuous variables were compared using a paired Student's t-test. Results comparing more than two groups were analyzed by ANOVA followed by Kruskal-Wallis statistics. Values were considered significant at *P *< 0.05. Resulting data files from the gene expression profiling experiments were analyzed using RMA from BIOconductor. All statistical analysis and graphs were constructed using GraphPad software.

## Results

### Decitabine increases MHC I and CTA expression

**Figure 1:**
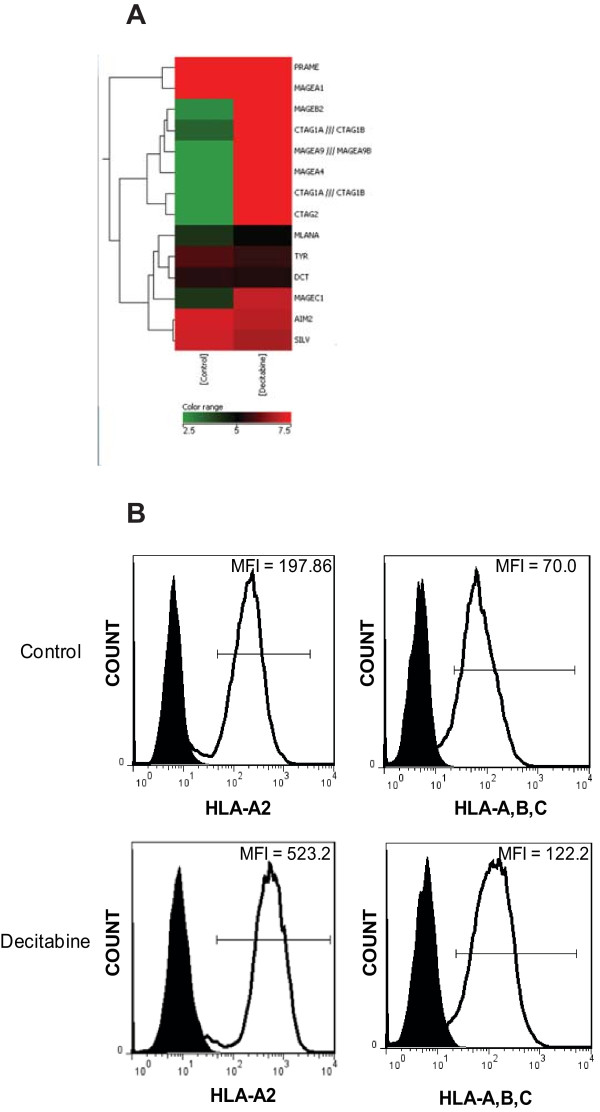
**Treatment of human glioma with decitabine up-regulates cancer-testes antigens and MHC I**. **A) **Total RNA was isolated from U-251MG glioma cells treated with decitabine or vehicle control and subjected to global gene expression classification using Affymetrix human U133 Plus 2.0 microarray chips. **B) **Decitabine-treated and untreated U-251MG glioma cells were stained with α-HLA-A, B, C and α-HLA-A2. Representative flow cytometric analysis data are shown here. Similar results were seen in two independent experiments.

We previously demonstrated that many melanoma associated antigens (MAA) are expressed by glioma cells, but at distinctly lower levels compared with melanoma cells [32]. To identify epigenetically silenced genes in glioma cells, we treated cells with the DNA methyltransferase inhibitor, decitabine, and then performed global gene expression profiling. Many CTA and major histocompatibility complex (MHC class I) antigens can exhibit methylation in their promoters, thus influencing their protein expression at an epigenetic level. As shown in Figure 1A, an unbiased microarray screening ranked a significant number of CTA as being highly up-regulated after decitabine treatment. These data included a 58-376 fold increase in NY-ESO-1 and other related CTA (Table 1). Multiple genes from the MHC and death receptor family were also upregulated (Additional File 1). To confirm changes in the protein expression of MHC, we stained cells with monoclonal antibodies to pan class I (HLA-A, B, C) as well as to HLA-A2 specifically. Decitabine treatment resulted in increased surface expression to MHC-class I molecules and to HLA-A2 (Figure 1B).

**Table 1:** 

Gene Symbol	Gene Title	Fold Induction
**PRAME**	Preferentially expressed antigen in melanoma	1.01
**MAGE-A1**	Melanoma antigen Family -A1	1.39
**MAGE-B2**	Melanoma antigen Family-B2	92.92
**CTAG1A///CTAG1B**	Cancer/testis antigen 1A/1B (NY-ESO-1)	376.59
**MAGE-A9**	Melanoma antigen Family-A9	83.45
**MAGE-A4**	Melanoma antigen Family-A4	248.02
**CTAG1A///CTAG1B**	Cancer/testis antigen 1A/1B (LAGE-2, NY-ESO-1)	57.90
**CTAG2**	Cancer/testis antigen-2 (LAGE-1, CAMEL)	654.87
**MLANA**	Melan-A/MART-1	1.86
**TYR**	Tyrosinase (oculocutaneous albinism 1A)	0.82
**DCT**	Dopachrome tautomerase (TRP-2)	0.94
**MAGE-C1**	Melanoma antigen Family-C1	7.03
**AIM-2**	Absent in melanoma-2	0.82
**SILV**	Silver homolog (gp100)	0.78

*(log_2 _of normalized gene expression values)

### Decitabine increases the expression of NY-ESO-1 on established glioma cells and primary glioblastoma cells obtained from surgical resection

**Figure 2:**
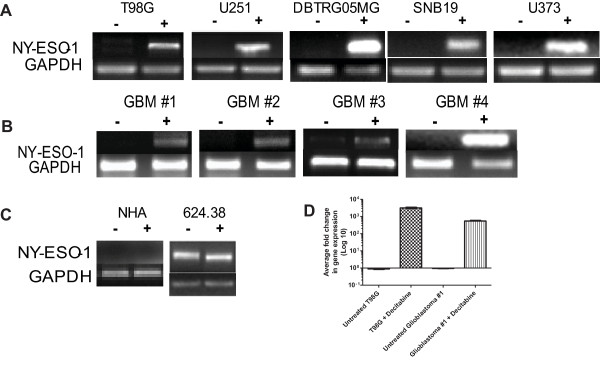
**Up-regulated expression of NY-ESO-1 in human glioma-derived cell lines and primary cell lines treated with decitabine**. **A) **Representative data for established glioma cell lines; **B) **primary glioblastoma cells obtained from surgical resection; or **C) **normal human astrocytes (NHA) and a NY-ESO-1^+ ^human melanoma line, 624.38. Only decitabine-treated glioma cells demonstrated expression of NY-ESO-1. **D) **Using SYBR green DNA labeling and quantitative PCR primers specific for NYESO-1 and GAPDH, the cDNA was amplified using a real-time PCR protocol. The relative fold change in gene expression is graphed showing an increase average fold change in treated T98G and glioblastoma #1 cells compared to untreated (***p = 0.0001 and **p = 0.007). Similar results were seen in three replicate experiments.

To confirm our microarray results, conventional RT-PCR and quantitative RT-PCR were used to assess NY-ESO-1 expression in established glioma cell lines and primary glioblastoma cell explants placed into culture immediately after surgical resection (Figure 2). Multiple glioma cell lines allowed us to assess whether the induction of NY-ESO-1 expression was a consistent feature of decitabine treatment. Results showed a uniform up-regulation of NY-ESO-1 in all decitabine-treated cell lines, with some variation in levels of expression (Figure 2A). Decitabine-treated primary cell lines also showed uniform up-regulation of NY-ESO-1 expression (Figure 2B). However, such up-regulation of NY-ESO-1 did not occur in non-malignant, normal human astrocytes (NHA), alleviating potential concerns that systemic administration of decitabine will up-regulate CTA on normal brain cells (Figure 2C). An NY-ESO-1^+^HLA-0201^+ ^melanoma cell line (624.38) was used as a positive control. The high endogenous expression of NY-ESO-1 was not changed with decitabine treatment (Figure 2C). To quantify the induction of NY-ESO-1 after decitabine treatment, real-time PCR was performed, which correlated with the results obtained with conventional RT-PCR (Figure 2D). Compared to the untreated control cells, the decitabine-treated T98G and glioblastoma #1 cells showed average log fold increases in the gene expression of NY-ESO-1 (3155.12 and 544.29, respectively) both of which were statistically significant (*p *= 0.0001 and 0.0075).

**Figure 3:**
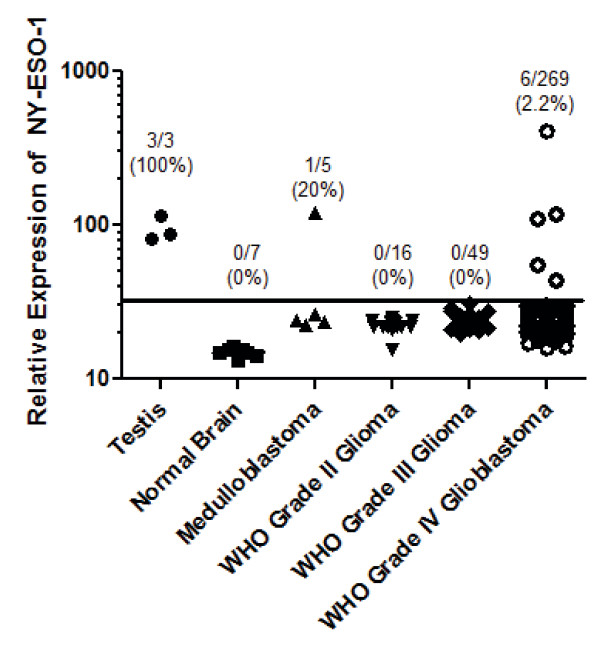
**Expression of NY-ESO-1 in human brain tumor tissues**. A large panel of normal tissues and brain tumors of varying grades were subjected to global gene expression profiling using Affymetrix U133 2.0 chips. The relative expression of NY-ESO-1 was normalized and tested. Dots plotted underneath the black line are considered to have background relative expression levels.

To evaluate the baseline expression of NY-ESO-1 in heterogeneous human brain tumor tissues, a large panel of normal tissues and brain tumors of varying grades were subjected to global gene expression profiling using Affymetrix U133 2.0 chips. NY-ESO-1 was not expressed in lower-grade gliomas (WHO Grades II-III). However, significantly elevated expression was detectable in a small percentage of medulloblastoma and glioblastoma (WHO Grade IV) tumor specimens. Such expression was comparable to the expression of NY-ESO-1 observed in normal testes, the only non-malignant, postnatal tissue that normally expresses this CTA (Figure 3). These data clarify the frequency and degree of expression of NY-ESO-1 in heterogeneous patient samples.

### Immunosensitization of human glioma after decitabine treatment

**Figure 4:**
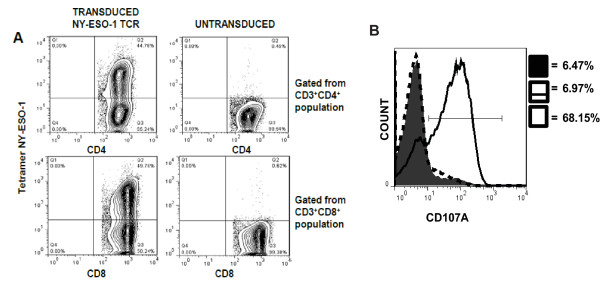
**Retroviral transduction of PBMC with a cloned NY-ESO-1 specific T cell receptor allows for T cell recognition of decitabine-treated human T98 glioma cells**. Normal donor peripheral blood mononuclear cells (PBMCs) were activated with OKT-3 at a concentration of 50 ng/mL for two days and then harvested for transduction. **A) **PBMCs were transduced with retroviral constructs encoding a HLA-A*0201-restricted NY-ESO-1 T cell receptor, and stained with fluorescent mAbs and tetramer NY-ESO-1 percentage. **B) **Representative flow cytometric analysis of CD107A of decitabine-treated and untreated T98G glioma cells (solid filled, NY-ESO-1 specific T cells alone; dashed line, NY-ESO-1 specific T cells co-cultured with untreated T98G glioma cells; solid line, NY-ESO-1 specific T cells co-cultured with decitabine-treated T98G glioma cells). Golgi-plug was added to co-cultures and stained with CD107A before incubation. The lymphocytes were stained with antibodies to CD3, CD4, and CD8 and subsequently fixed. The experiment was repeated three times with similar findings.

To evaluate whether up-regulated expression of NY-ESO-1 and MHC I sensitized these tumor cells to T cell recognition, we expressed an NY-ESO-1 specific T-cell receptor clone 1G4 in normal human PBMCs utilizing a retroviral transduction system [30]. Within the transduced CD3^+^CD8^+ ^T-cell population of PBMCs, 56% of cells expressed TCRVβ13.1, a TCR known to be expressed by the clone 1G4-α95:LY NY-ESO-1 TCR [30] (Additional File 2). Untransduced cells showed only small frequencies of a TCRVβ13.1^+ ^T cell population. Tetramer staining revealed that nearly 50% of the CD3^+^CD8^+ ^population was NY-ESO-1 specific (Figure 4A). Tetramer staining also showed that approximately 45% of the CD3^+^CD4^+ ^population was NY-ESO-1 specific.

**Figure 5:**
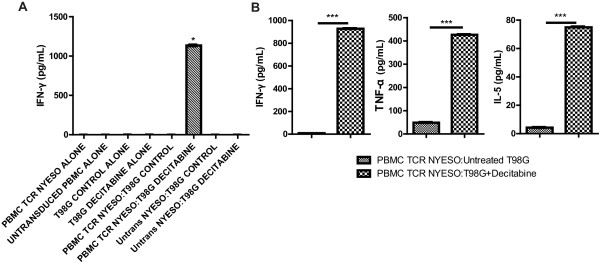
**Elevated cytokine secretion of NY-ESO-1 TCR-transduced PBMCs co-cultured with decitabine-treated T98G glioma cells**. T98 glioma cells were treated with or without decitabine and then co-cultured with NY-ESO-1 TCR-transduced T cells. Cell-free supernatants were tested for cytokine secretion by IFN-γ ELISA and cytometric bead array. **A) **Data from the IFN-γ ELISA is depicted (*p <*0.05). **B) **Concentrations of Th1/Th2 cytokines IFN-γ, TNF-α, and IL-5 (pg/mL) in co-cultured samples are depicted (***p < 0.001). Results shown are representative findings from over 5 replicate experiments.

To evaluate the functional recognition of glioma cells by NY-ESO-1 specific T cells, the cells were co-cultured and then stained for intracellular expression of CD107A (LAMP-1). The cell surface mobilization of CD107A has been directly linked to CTL lytic granule release and target cell death [33]. In a representative study that has been repeated at least 3 times, NY-ESO-1 specific CD8^+ ^T cells, gated from the CD3^+^CD8^+ ^population, exhibited a 7% or a 68% expression of CD107A when co-cultured with control or decitabine-treated T98 glioma cells, respectively (Figure 4B). NY-ESO-1 specific CD4^+ ^T cells, gated from the CD3^+^CD4^+ ^population, did not respond to the co-cultures (data not shown). NY-ESO-1 specific T cells co-cultured with an HLA-A*0201^+^, NY-ESO-1^+ ^melanoma line (624.38) exhibited a 65% expression of CD107A (data not shown). To assess the release of cytokines by NY-ESO-1 specific T cells, we used the cell-free supernates of overnight co-cultures for ELISA and CBA. As shown in Figure 5A, significantly elevated concentrations of IFN-γ (1133.9 pg/ml) were detected when NY-ESO-1 specific T cells were co-cultured with decitabine-treated T98 glioma cells, whereas, co-culture with untreated glioma cells was below the level of detection. Co-culture of untransduced PBMCs with control or decitabine-treated glioma cells also did not elicit the release of detectable IFN-γ. Using cytometric bead arrays, we were able to detect multiple Th1/Th2 cytokines simultaneously secreted by NY-ESO-1 specific T cells. Significant differences in the concentrations of IFN-γ, TNF-α, and IL-5 were observed only when NY-ESO-1 specific T cells were co-cultured with decitabine-treated T98 glioma cells (Figure 5B; *p *< 0.0001). Small differences were also seen with IL-2 and IL-4, but not IL-10 (data not shown). Thus, treatment of T98 glioma cells with decitabine results in significantly enhanced NY-ESO-1 specific T cell recognition and secretion of Th1-type cytokines along with IL-5.

### Decitabine sensitizes cells to immune-mediated cell death via a Fas/Fas Ligand pathway

**Figure 6:**
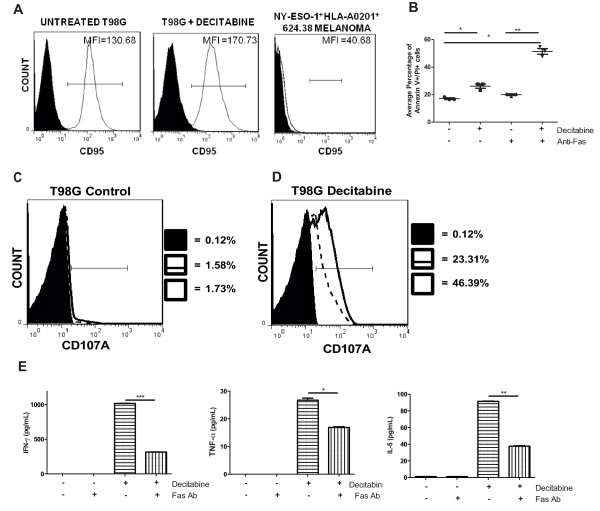
**Decitabine sensitizes glioma cells to an immune-mediated cell death via the Fas/FasL pathway**. **A) **Decitabine-treated and untreated T98G cells were stained with an anti-Fas antibody and analyzed by flow cytometric analysis. **B) **T98G cells were treated as described previously and stained with an agonistic-Fas antibody (clone CH-11) to stimulate apoptosis for 24 hr. Results depict the average percentage of late cell death in untreated alone, decitabine-treated alone, untreated with agonistic mAb, and decitabine-treated with agonistic mAb. Decitabine-treated alone versus decitabine-treated with antibody (*P *= 0.0049). Untreated alone versus untreated with antibody (*P *= 0.0215). Experiments were conducted in triplicate. **C) **Representative flow cytometric analysis of CD107A staining from NY-ESO-1 TCR transduced T cells co-cultured with untreated T98G glioma cells, with or without the addition of an antagonistic-Fas antibody (clone ZB4) to block apoptosis (solid filled, NY-ESO-1 specific T cells alone; solid line, NY-ESO-1 specific T cells co-cultured with untreated T98G glioma cells; dashed line, NY-ESO-1 specific T cells co-cultured with untreated T98G glioma cells with antagonistic-Fas antibody. NY-ESO-1 TCR transduced T cells were gated from the CD8 population. **D) **Representative flow cytometric analysis of CD107A staining from NY-ESO-1 transduced T cells co-cultured with decitabine-treated T98 glioma cells, with or without the addition of an antagonistic-Fas antibody (clone ZB4) to block apoptosis (solid filled, NY-ESO-1 specific T cells alone; solid line, NY-ESO-1 specific T cells co-cultured with decitabine-treated T98G glioma cells; dashed line, NY-ESO-1 specific T cells co-cultured with decitabine-treated T98G glioma cells with antagonistic-Fas antibody. **E) **CBA was performed on cell-free supernatant of samples from Figure 6C. Th1/Th2 cytokine concentrations IFN-γ, TNF-α, and IL-5 (pg/mL) are depicted. (*p < 0.05; ** *p *< 0.01; NS, not significant).

To test whether decitabine treatment sensitized glioma cells to distinct immune-mediated death receptor pathways, we assessed expression of TNFR, TRAIL-DR4, TRAIL-DR5, and Fas on T98 glioma cells by flow cytometric analyses. No changes in the expression of TNFR, TRAIL-DR4, or TRAIL-DR5 were observed after decitabine treatment (data not shown). Small, but detectable increases in the mean fluorescence intensities (MFI) of Fas (CD95) were observed after decitabine treatment (Figure 6A). Furthermore, addition of an agonistic Fas mAb to human T98 glioma cells enhanced tumor cell death when pre-treated with decitabine (Figure 6B). Staining of T98G glioma cells with Annexin V and propidium iodide (PI) indicated the average percentages of late apoptotic cells at 17%, 26%, 20%, and 51% in untreated, untreated with Fas mAb, decitabine-treated alone, and decitabine-treated with Fas mAb, respectively (Figure 6B). One-way ANOVA statistical testing showed a significant difference between the control and Fas-antibody treated groups (*p *< 0.01). There was also a significant difference observed within untreated and decitabine-treated groups with the addition of the agonistic Fas mAb (*p = 0.0215 and **p = 0.0049). Such effects on Fas-mediated apoptosis were consistently observed at timepoints as early as 4-6 hours and as late as 4 days (data not shown).

Because decitabine pre-treatment sensitized glioma cells to Fas mAb mediated cell death with an agonistic mAb, we also tested whether such death receptor pathways were functionally important for NY-ESO-1 specific T cell recognition and killing of glioma cells. Untreated T98G glioma cells, blocked with a Fas antagonistic antibody, showed no difference in CD107A expression compared to untreated T98G glioma cells co-cultured with NY-ESO-1 specific T cells (Figure 6C). However, decitabine-treated T98G glioma cells, blocked with a Fas antagonistic antibody, showed dramatic decreases in CD107A expression levels compared to decitabine-treated T98G glioma cells co-cultured with NY-ESO-1 specific T cells (Figure 6D). The NY-ESO-1 specific T cells alone (negative control) showed minimal release of CD107A expression. To address the effect of blocking the Fas pathway on cytokine release, supernatants derived from NY-ESO-specific T cells co-cultured with T98G cells were tested in cytokine bead arrays. Addition of the antagonistic Fas mAb to decitabine-treated cells largely inhibited the entire induction of Th1-type cytokine release (IFN-γ and TNF-α) by NY-ESO-1 specific T lymphocytes (Figure 6E). IL-10 concentrations appeared to decrease but were not as prominent as the other Th1/Th2 cytokines (data not shown). IL-5 concentrations were almost completely inhibited as well.

## Discussion

One of the major challenges of developing immunotherapeutic techniques that target cancer/testis antigens, such as NY-ESO-1, is their low and variable baseline expression in solid tumors. We demonstrated that baseline, untreated glioma cells do not express detectable levels of NY-ESO-1. However, treatment with 1 μM of decitabine for 48 hr significantly induced the expression of NY-ESO-1 mRNA expression, as shown by conventional and quantitative RT-PCR. Such data confirms the recent work by Natsume, et al., who demonstrated that the CTA upregulation was coordinately associated with decreased promoter methylation [21]. Microarray data suggest that approximately 2% of glioblastomas express NY-ESO-1. However, NY-ESO-1 expression can be induced quickly with decitabine treatment, indicating epigenetic regulation likely is responsible for the normal silencing of NY-ESO-1. Increases in NY-ESO-1 expression resulted in NY-ESO-1 specific T cell recognition and killing of human glioma cells. The functional NY-ESO-1 specific T cell recognition of decitabine-treated glioma cells was comparable to the recognition of a human melanoma cell line known to express high levels of NY-ESO-1 [15,30]. Together, such data suggest that adjuvant treatment with agents that modify DNA methylation could potently synergize with targeted immunotherapy for cancer.

NY-ESO-1 is a cancer/testis antigen that is considered one of the most immunogenic members in the cancer testes antigen family. NY-ESO-1 is frequently expressed in melanomas but not typically expressed in gliomas, as are other members of the CTA family [10,13,14,34,35]. Our results showed that NY-ESO-1 was exclusively expressed in decitabine-treated glioma cells, but not expressed in untreated glioma cells or normal human astrocytes (Figure 2). This is important because it suggests that decitabine will up-regulate the relative expression of NY-ESO-1 only in tumor cells, therefore alleviating concerns that decitabine treatment *in vivo *could cause autoimmunity in patients undergoing NY-ESO-1 T cell adoptive transfer. In support of this, Robbins *et al*. demonstrated that the adoptive transfer of NY-ESO-1 TCR-transduced T cells in patients with metastatic melanoma and synovial cell sarcoma did not result in any significant toxicity, suggesting that targeting CTAs using TCR-transgenic lymphocytes mediates the regression of established tumors without damaging normal tissues [15]. Thus, the effects of decitabine appear to be tumor specific, inducing the expression of multiple CTAs, such as NY-ESO-1, that can be potentially used for immunotherapeutic targeting.

Recent studies have shown that immunotherapy with antigen specific T cells can recognize tumor cells that express the corresponding antigen, but the apoptotic signaling system and survival pathways for such cells remain dysfunctional [36-38]. Weller *et al*. suggested that the endogenous apoptosis pathway can be induced in activated human malignant gliomas [39]. The immunosensitization of death receptor pathways in tumor cells is one mechanism by which cancer immunotherapy death receptor activation by T cells is relatively selective to tumor cells compared to non-malignant cells as previously described [40,41]. However, evasion of apoptosis is a major characteristic in most cancers, including glioblastomas. This can be caused by the dysfunction in signaling downstream of Fas and possibly other death receptors [38]. Fas/FasL is an extrinsic death pathway that induces apoptosis upon trimerization of Fas after binding to FasL leading to the recruitment of Fas-associated death domain (FADD). As most glioblastoma cells are resistant to apoptosis induced by Fas ligand, induction of apoptosis through this pathway in gliomas will require sensitization by other means [42,43]. Our results suggest that treatment with decitabine could re-establish the functionality of this signaling system and sensitize cells to an immune-mediated cell death by the Fas/FasL pathway.

Degranulation is an essential step in the process of perforin-granzyme mediated killing and is critical for rapid lytic function mediated by responding antigen-specific CD8^+ ^T cells. Lysosomal-associated membrane protein-1 (LAMP-1 or CD107A) is a sensitive marker of cytotoxic CD8^+ ^T cell degranulation [33,44] that increases dramatically upon T cell recognition of antigen-expressing target cells. Our present results showed that the expression of CD107A of decitabine-treated glioma cells blocked with an antagonistic Fas mAb decreased to almost 50% compared to decitabine-treated cells. A previous study demonstrated that the use of an antagonistic Fas mAb was specific to the Fas receptor [45]. Our results strongly suggest that by blocking the Fas/FasL pathway, there is a decrease in the efficacy of targeting tumor cells by the cytotoxic CD8^+ ^T cells when treated with decitabine. A recent study suggested that rapid clearance of cancerous cells is most effective through the use of both the Fas/Fas Ligand and Perforin/Granzyme pathways due to their complementary, non-redundant killing mechanisms [36]. The present data therefore support the role of decitabine in sensitizing glioma cells to apoptosis by the Fas/FasL pathway.

## Conclusions

Our studies demonstrate that utilization of a demethylating agent, such as decitabine, could induce the expression of CTA such as NY-ESO-1 on glioma cells, which in turn can act as effective target molecules for T cell mediated therapy. Induction of this antigen, along with an increased expression of MHC I, and upregulation of proinflammatory cytokines may sensitize tumor cells to tumor specific T cell-mediated cell injury. To translate such findings into patients, we have already planned a first-in-human Phase I clinical trial. In this trial, patients with recurrent glioblastoma will receive pre-operative decitabine (Dacogen^**®**^), followed later by the local adoptive transfer of NY-ESO-1 TCR-transduced T lymphocytes through a Rickham reservoir placed during surgery. Given the short half-life of decitabine and long-lasting effects of the induced NY-ESO-1 expression, we anticipate that we will have a window of opportunity to infuse such T cells without any drug suppression. Overall, our studies indicate that decitabine can aid in the effectiveness of cellular therapy with T cells targeting NY-ESO and provide and eliminate concerns that they will also target normal brain cells.

## Abbreviations

(TRA): Tumor-rejection antigens; (CTAs): Cancer testis antigens; (PBMC): Peripheral blood mononuclear cells; (cDNA): complimentary deoxyribonucleic acid; (RT-PCR): reverse transcription-polymerase chain reaction; (RN): Retronectin; (TCR): T cell receptor; (ELISA): enzyme-linked immunosorbent assay; (CBA): cytokine bead array; (mAb): monoclonal antibody; (PI): propidium iodide; (FasL): Fas ligand

## Competing interests

The authors declare that they have no competing interests.

## Authors' contributions

VK carried out in vitro treatment of glioblastoma cells, generation of NY-ESO-1 specific lymphocytes, flow cytometric, conventional and quantitative RT-PCR analysis, ELISA, cytometric bead array, apoptotic studies and drafted the manuscript. WK carried out initial experiments that led to the microarray results. RCK designed the transduction protocol and helped generate NY-ESO-1 specific lymphocytes. MD participated in treatment of primary cell cultures as well as RT-PCR analysis. AE and SN performed statistical analysis as well as made substantial contribution to the interpretation of microarray data. AR and LML participated in design of the study as well as editing of the manuscript. RMP conceived and designed the study, wrote and guided the editing of the manuscript. All authors read and approved the final manuscript.

## Supplementary Material

Additional file 1 **Treatment of human glioma with decitabine up-regulates MHC I**. Total RNA was isolated from U251 glioma cells treated with decitabine or vehicle control and subjected to global gene expression classification using Affymetrix human U133 Plus 2.0 microarray chips. Data was analyzed with dChip microarray software. Click here for file

Additional file 2 **Flow cytometric characterization of NY-ESO-1 TCR-transduced PBMC**. Normal donor peripheral blood mononuclear cells (PBMCs) were activated with OKT-3 at a concentration of 50 ng/mL for two days and then harvested for transduction. PBMCs were transduced with retroviral constructs encoding an HLA-A201-restricted NY-ESO-1 T cell receptor, stained with multi-colored antibodies to CD3, CD8, and TCRV β13.1, and finally analyzed by flow cytometry to estimate the efficiency of transduction. Click here for file
