# Supplementary figures and images for: Decitabine immunosensitizes human gliomas to NY-ESO-1 specific T lymphocyte targeting through the Fas/Fas Ligand pathway
Source: J Transl Med. 2011 Nov 7;9:192. doi: 10.1186/1479-5876-9-192 (PMC3229551; doi:10.1186/1479-5876-9-192)

U251 Control  
U251+Decitabine

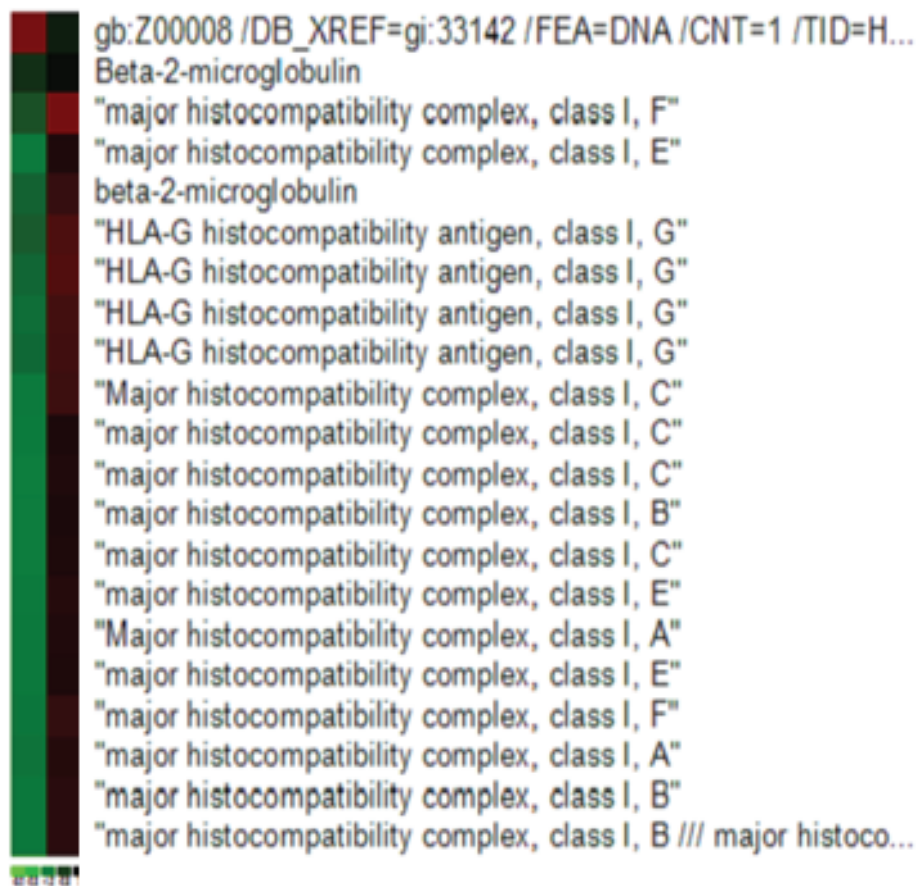

Supplement: Additional file 1 — Treatment of human glioma with decitabine up-regulates MHC I. Total RNA was isolated from U251 glioma cells treated with decitabine or vehicle control and subjected to global gene expression classification using Affymetrix human U133 Plus 2.0 microarray chips. Data was analyzed with dChip microarray software. [file 1479-5876-9-192-S1.PDF]

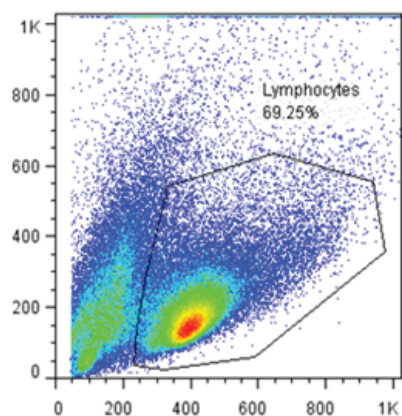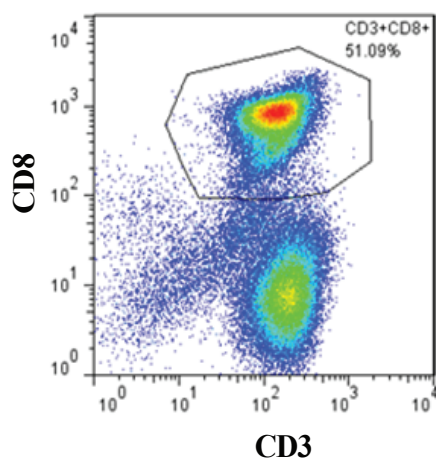

### TRANSDUCED

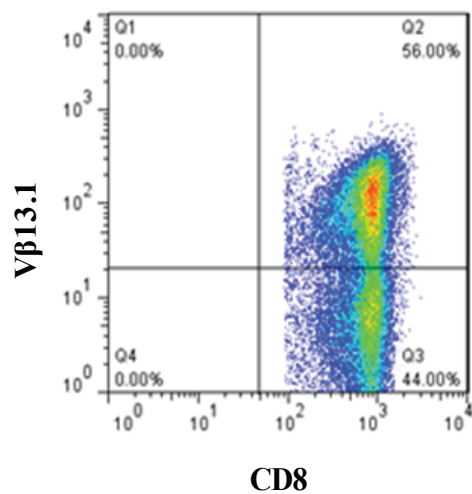

### UNTRANSDUCED

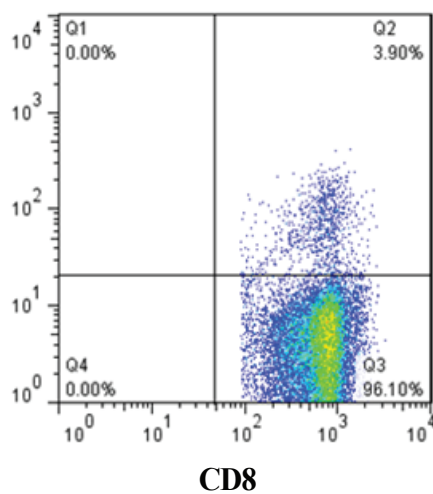

### ISOTYPE

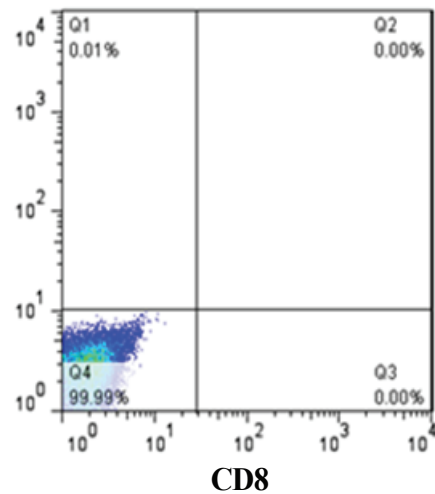

Supplement: Additional file 2 — Flow cytometric characterization of NY-ESO-1 TCR-transduced PBMC. Normal donor peripheral blood mononuclear cells (PBMCs) were activated with OKT-3 at a concentration of 50 ng/mL for two days and then harvested for transduction. PBMCs were transduced with retroviral constructs encoding an HLA-A201-restricted NY-ESO-1 T cell receptor, stained with multi-colored antibodies to CD3, CD8, and TCRV β13.1, and finally analyzed by flow cytometry to estimate the efficiency of transduction. [file 1479-5876-9-192-S2.PDF]
